# Medical Chemistry

**Published:** 1890-05

**Authors:** 


### Book Reviews.

MEDICAL CHEMISTRY.—By Elias H. Bartley, M. D., Professor of Chemistry, Long Island Medical College. Second edition. pp. 425. Philadelphia. P. Blakiston, Son & Co., 1890.

The second edition of this work comes to us with no lengthy preface, making excuses for its publication, or attempting to explain its purposes. In truth no such preface is needed; the book speaks for itself, and we think fills a want in clinical literature.

The book is admirably suited to the student of medicine. It is well printed, well indexed, and contains a useful glossary of unusual chemical terms.

The author devotes the first portion of the volume to chemical physics, touching on heat, light, electricity and crystallography, advancing only such facts and principles as are absolutely essential to the student before beginning chemistry proper.

Part second is devoted to chemical notation and nomenclature, and in the space of twenty pages the author makes this part of his subject clear, and so arranges it that it can be easily comprehended by the student.

Part third treats of inorganic chemistry, taking up the more important chemical elements, giving their occurrence, preparation and properties, together with tests for the same, and their physiological action.

Part fourth takes up organic chemistry, and in as short space as possible goes into the chemistry of Benzol, the alcohols, the organic acids, the Ptomaines, the Glucocides &c. &c., and closes the work with a discussion of the urine, normal and pathological. L. H. J.

